# PROTOCOL: Effects of nutritional interventions during pregnancy on birth, child health, and development outcomes: A systematic review of evidence from low‐ and middle‐income countries

**DOI:** 10.1002/cl2.1019

**Published:** 2019-07-13

**Authors:** Zohra S. Lassi, Aamer Imdad, Deepika Ranjit, Gamael Saint Saint Surin, Rehana A. Salam, Zulfiqar A. Bhutta

**Affiliations:** ^1^ Robinson Research Institute University of Adelaide Adelaide South Australia Austalia; ^2^ SUNY Upstate Medical University Syracuse New York; ^3^ Department of Pediatrics Aga Khan University Karachi Sindh Pakistan; ^4^ Global Child Health The Hospital for Sick Children Toronto Ontario Canada

## 1 BACKGROUND

### 1.1 The problem, condition or issue

Optimal nutrition plays a crucial role before, during and after pregnancy (Alfaradhi & Ozanne, 2011; Black et al., 2013; Ota, Hori, Mori, Tobe‐Gai, & Farrar, 2015). Poor maternal nutritional status is a risk factor for serious foetal complications and the outcomes for the neonate, including intrauterine growth restriction, stillbirth, low birth weight, preterm birth, increased risk of neonatal infections, neonatal hypothermia and neonatal death (Ahmed, Hossain, & Sanin, 2012; Black et al., 2013). Moreover, women who are undernourished at the time of conception have a higher risk of obstructed labour, preeclampsia, anaemia and mortality when compared with healthy women (Christian, Mullany, Hurley, Katz, & Black, 2015; Zerfu, Umeta, & Baye, 2016).

The prevalence of maternal malnutrition is higher in low‐ and middle‐income countries (LMICs) when compared with high‐income countries (Black et al., 2013). Malnutrition refers to a group of nutritional disorders that include micronutrient deficiencies, undernutrition and overweight/obesity. Maternal under‐nutrition is typically defined by a body mass index (BMI) less than 18.5 kg/m^2^, while overweight is classified as BMI ≥25 kg/m^2^ and obesity as BMI ≥30 kg/m^2^. The double burden of malnutrition is the coexistence of undernutrition and overweight and obesity, which has also been found to be highly prevalent in LMICs (Kimani‐Murage et al., 2015) due to diets that chronically lack diversity and infections and/or chronic disease that could contribute to deficiencies by directly inhibiting nutrient absorption.

The prevalence of maternal undernutrition ranges from 10 to 19% in LMICs, with variation by region and by country (Black et al., 2013). In addition, >10% of women aged 15–45 living in LMICs have heights (i.e., maternal stunting defined as maternal height <145 cm) that are considerably below the average (Black et al., 2013). The prevalence of low BMI in adult women is >20% in Sub‐Saharan Africa and South‐Central and Southeastern Asia (Black et al., 2013). Some individual countries fare worse than others. For example, in India, the prevalence of undernutrition among women of reproductive age (WRA) reaches almost 40% (Black et al., 2013). In 2014, about 1.9 billion adult people worldwide were found to be overweight, a prevalence that surpassed that of underweight, which constituted about 462 million people. In addition, >600 million were reported to be obese (World Health Organization, 2017). The prevalence of obesity is higher in the Americas and the Caribbean when compared to Africa, but overall, rates of overweight and obesity are rising globally, a situation that mimics that in high‐income countries and may be reflective of changing food environments (Black et al., 2013; WHO, 2017).

Both maternal undernutrition and overnutrition can have adverse effects before, during and after pregnancy (Kimani‐Murage et al., 2015). Here add something about maternal undernutrition being related to low birthweight. Maternal undernutrition throughout pregnancy has also been associated with long‐term health issues for the infant, such as obesity, diabetes mellitus, hypertension and cognitive dysfunction (Crispi, Miranda, & Gratacós, 2018; Maršál, 2016). In addition, low birth weight has been associated with increased risk of death from coronary heart disease and stroke in adulthood (Crispi et al., 2018). Malnutrition or inadequate dietary intake during pregnancy can expose the foetus to a harsh environment, which forces the foetus to adapt. However, this adaptation can lead to permanent changes in function and structure that can later lead to chronic diseases in adult life (Crispi et al., 2018; Maršál, 2018). Maternal obesity has also been associated with a higher risk of stillbirth and congenital abnormalities (Alfaradhi & Ozanne, 2011; Stothard, Tennant, Bell, & Rankin, 2009). In addition, obesity during pregnancy is associated with an increased risk of foetal macrosomia (Catalano & DeMouzon, 2015), which could lead to obstructed labour, and preterm birth, which is a major risk factor for infant mortality (Meehan, Beck, Mair‐Jenkins, Leonardi‐Bee, & Puleston, 2014). This review will focus on macronutrient supplementation during pregnancy. Micronutrient supplementation is being evaluated in a separate Campbell review of this series.

### 1.2 The intervention

Several macronutrient supplementation interventions have been proposed to address maternal malnutrition especially in LMICs including balanced energy supplementation, food provision and distribution and dietary intervention to prevent maternal obesity (Bhutta et al., 2013; Imdad & Bhutta, 2012).

In LMICs, diets often lack foods rich in macronutrients and micronutrients that are typically found in meat, poultry and fish (Gibson & Hotz, 2018). Therefore, it is important to increase the availability of macronutrients and micronutrients by promoting and introducing diverse crops, integrating farming systems with small livestock, promoting fish farming and promoting better food storage (Gibson & Hotz, 2018). In addition, this intervention includes supplementation, which is designed to supply pregnant women in LMICs with multiple micronutrients (Allen, De Benoist, Dary, & Hurrell, 2006; Gibson & Hotz, 2018; Zerfu et al., 2016). Such interventions have found to be positively related to a reduced risk of maternal anaemia, preterm birth and low birth weight in a single study in Ethiopia (Zerfu et al., 2016).

A balanced energy protein (BEP) supplement is a macronutrient food‐based supplement where proteins provide <25% of total energy content (Imdad, 2012). BEP supplements, therefore, come in several forms. For example, a study from India provided supplements that consisted of dehusked sesame cake, jaggery and oil containing 30 g of proteins and 417 kcal energy for undernourished pregnant women (Girija, Geervani, & Rao, 1984). In another study from The Gambia, undernourished pregnant women were given daily supplements of high energy biscuits made with roasted nuts, rice flour, sugar and groundnut oil as supplements that contained 4,250 kJ energy, 22 g of proteins, 56 g fat and vitamins and minerals (Ceesay et al., 1997).

Two previous reviews have demonstrated the positive association of BEP interventions with pregnancy outcomes, such as a reduced risk of stillbirth and small for gestational age babies and increase of birth weight (Imdad, 2012; Ota et al., 2015).

Food distribution programmes provide low‐income and undernourished pregnant and nonpregnant women and children with access to supplemental nutritious foods and often nutrition education (Baqui et al., 2008; Heaver, 2002; Kapil, Chaturvedi, & Nayar, 1992). These programmes are typically run by local or international organisations. For example, India has the Integrated Nutrition and Health programme, which is a non‐governmental organisation‐based programme that is implemented together through CARE‐India and the Indian government (Baqui et al., 2008). This programme educates pregnant women alongside the provision of healthcare services and supplementary nutrition, with the aim of increasing knowledge about maternal and newborn care. The long‐term goal of reducing neonatal mortality (Baqui et al., 2008; Kapil, 2002). India also has the Tamil Nadu Integrated Nutrition Programme (TINP), which is implemented by the state government of Tamil Nadu and supported by the World Bank. TINP aims to reduce maternal and child malnutrition through the use of a Community Nutrition Centre that makes supplementary nutrition available to pregnant women and children in villages (Heaver, 2002). In Bangladesh, the nutrition‐focused Maternal, Neonatal, and Child Health programme supports pregnant women by providing several cross‐cutting services such as counselling on nutrition and health, micronutrient supplementation and weight‐gain monitoring (Nguyen et al., 2017).

As noted above, obesity during pregnancy is associated with a host of maternal and foetal complications such as pre‐eclampsia, caesarian birth, macrosomia and congenital malformations (Dodd, Crowther, & Robinson, 2008; Muktabhant, Lawrie, Lumbiganon, & Laopaiboon, 2015). Several behavioural interventions, including dietary control and exercise, have been found to be positively related to a reduced risk of macrosomia, caesarean delivery and gestational weight gain (GWG; Catalano & DeMouzon, 2015; Dodd et al., 2008; Guelinckx, Devlieger, Mullie, & Vansant, 2010; Muktabhant et al., 2015; Renault et al., 2014). Interventions can vary, and could include light to moderate‐intensity exercise, strength training, stretching and relaxation exercises to prevent excessive weight gain (Nascimento, Surita, Parpinelli, Siani, & Pinto e Silva, 2011) or combined dietary control and exercise interventions whereby diet counselling and advice is paired with exercise. However, in this review we will only focus on dietary interventions to prevent maternal obesity.

### 1.3 How the intervention might work

BEP supplementation is used to help undernourished women achieve the recommended daily energy intake (Bhutta et al., 2013; Imdad, 2012). Currently, there is strong evidence to support the benefits of BEP supplementation when compared with both high protein energy supplements and isocaloric supplements (Imdad, 2012; Ota et al., 2015). Evidence from a Cochrane review has linked BEP supplementation to a reduction in stillbirths, small for gestational age births and improvement in birth weight (Ota et al., 2015). However, no significant impact on preterm birth or neonatal death was observed (Ota et al., 2015).

Food distribution programmes directly provide nutritious foods or supplements to vulnerable populations, including pregnant women. There is some evidence to support the targeting of programmes to pregnant women through the subsequent improvement in birth weight and reduction of infant mortality among infants of recipient mothers (Frith, Naved, Persson, & Frongillo, 2015). Often, programmes will provide pregnant women with healthy foods along with access to additional services, such as nutrition counselling. Counselling sessions may include information on the components of a healthy diet, the importance and consequences of poor nutrition, and food demonstrations, which provide women with the tools and knowledge necessary to maintain good antenatal health (Nguyen et al., 2017). Other interventions use community platforms, such as community health centres, to provide services such as immunisation, promotion of maternal and neonatal care, and distribution of food supplements. These strategies have have been shown to reduce neonatal deaths and improve maternal anaemia (Baqui et al., 2008; Leroy, Olney, & Ruel, 2016).

Lifestyle interventions that include dietary control, exercise and behavioural change have been associated with a reduced risk of excessive GWG and macrosomia and decreased risk of adverse pregnancy outcomes (Catalano & DeMouzon, 2015; Dodd et al., 2008; Guelinckx et al., 2010; Muktabhant et al., 2015; Renault et al., 2014). Moreover, lifestyle interventions for maternal obesity can be implemented using a combination of dietary control and physical activity (Renault et al., 2014) or diet and exercise and behavioural change alone (Muktabhant et al., 2015; Nascimento et al., 2011). Dodd et al. (2014) used a comprehensive antennal dietary and lifestyle counselling intervention for pregnant women in Australia. The intervention included exercise, home visits that provided dietary advice and behavioural strategies delivered by a registered dietician (Dodd et al., 2014).

### 1.4 Why it is important to do the review

**Figure 1:**
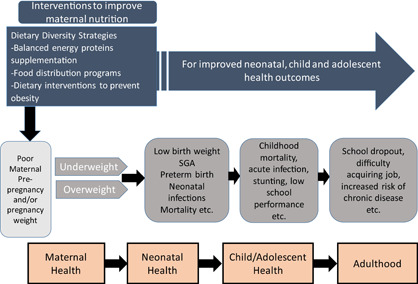
Framework on Maternal nutritional supplementation [Color figure can be viewed at wileyonlinelibrary.com]

Several reviews have been published that examine the impact of the interventions described above (Bhutta et al., 2013; Gibson & Hotz, 2018; Imdad & Bhutta, 2012; Muktabhant et al., 2015; Ota et al., 2015; Zerfu et al., 2016). However, most of these reviews focused on the efficacy of these interventions using randomised trials and did not address the question of effectiveness of large‐scale nutrition programmes. Studies of effectiveness are needed to understand whether an intervention will be impactful in a real‐world setting. Additional studies have been published recently (Devi et al., 2017; Dwarkanath et al., 2016; Huseinovic et al., 2017; Saville et al., 2018), indicating a need to update the systematic review evidence. Dietary interventions alone to prevent maternal obesity during pregnancy have not been reviewed previously. Therefore this review will make a first attempt to study its evidence. Furthermore, previous reviews did not assess the long term effects of these interventions during childhood. Taken together, this review will incorporate the latest evidence from RCTs and nonrandomized trials, and also assess the long term effects of maternal nutritional supplementation (Figure 1).

## 2 OBJECTIVES

This review aims to assess the effectiveness of nutritional interventions during pregnancy on maternal, neonatal and childhood outcomes. The specific objectives are to assess the effectiveness of following interventions during pregnancy on birth, child health and developmental outcomes:

1. BEP supplementation
2. Food distribution programmes
3. Dietary interventions to prevent maternal obesity

Each intervention will be assessed, analysed and reported separately.

## 3 METHODOLOGY

### 3.1 Criteria for including and excluding studies

#### 3.1.1 Types of study designs

1. Randomised controlled trials (RCTs), where participants were randomly assigned, individually or in clusters, to intervention and comparison groups. Cross‐over designs will be eligible for inclusion.
2. Quasi‐experimental designs, which include:
  (a) Natural experiments: studies where non‐random assignment is determined by factors that are out of the control of the investigator. One common type includes allocation based on exogenous geographical variation.
  (b) Controlled before‐after studies (CBA), in which measures were taken of an experimental group and a comparable control group both before and after the intervention. We also require that appropriate methods were used to control for confounding, such as statistical matching (e.g., propensity score matching, or covariate matching) or regression adjustment (e.g., difference‐in‐differences and instrumental variables).
  (c) Regression discontinuity designs; here, allocation to intervention/control is based upon a cut‐off score.
  (d) Interrupted time series (ITS) studies, in which outcomes were measured in the intervention group at least three time points before the intervention and after the intervention.

Two review authors will independently screen titles and abstracts of all retrieved references. We will retrieve the full‐text study reports for all citations that at least one review author considers potentially relevant. Two review authors will independently screen the full text articles and identify studies for inclusion, and identify and record reasons for exclusion of ineligible studies. We will include studies irrespective of whether measured outcome data are reported in a “usable” way. We will resolve any disagreement through discussion or, if required, we will consult a third review author. We will identify and excluded duplicates and collate multiple reports of the same study so that each study, rather than each report, is the unit of interest in the review. We will record the selection process in sufficient detail to complete a Preferred.

#### 3.1.2 Types of participants

This review will include healthy pregnant women of any age living in LMICs, as defined by the World Bank. Studies where women are recruited in the preconception period are eligible, given that women are followed throughout pregnancy. In this review, we will consider women who are undernourished (inadequate nutrition) and obese women who have no other co‐morbids.

#### 3.1.3 Types of interventions

This review will include the following interventions that target pregnant women:

1. BEP supplementation: Defined as a food supplement where proteins provide <25% of the total energy content (Imdad, 2012).
2. Food distribution programme: Food distribution programmes are defined by their direct provision of foods to recipients, who, in this case, are pregnant women. Eligible food distribution programmes could be locally or internationally led, and may or may not include elements of nutrition education.
3. Dietary interventions for prevention of maternal obesity: Eligible interventions for preventing or reducing maternal obesity include dietary control only.

Each intervention will be analysed separately and will not be compared to the other interventions listed here.

Comparison groups may include standard of care (routine diet).

#### 3.1.4 Types of outcome measures

This review will include studies that have the following primary and secondary maternal outcomes, foetal outcomes, newborn and child outcomes.

##### 3.1.4.1 Primary outcomes

- Maternal outcomes
  - BMI
- Foetal and newborn outcomes
  - Mortality:
    – Miscarriage defined as spontaneous expulsion of a human foetus before it is viable and especially between the 12th and 28th weeks of gestation
    – Stillbirth defined as baby born with no signs of life at or after 28 weeks' gestation.
    – Perinatal mortality is defined as: Stillbirth and deaths ≤7 days
    – Neonatal mortality (death <28 days)
- Child outcomes
  - Infant mortality (Deaths between 0 and 12 months)
  - Under‐five mortality (Deaths between 0 and 59 months.

##### 3.1.4.2 Secondary outcomes

- Maternal outcomes
  - Morbidity
    – Maternal mortality defined as the death of a woman while pregnant or within 42 days of termination of pregnancy, irrespective of cause.
    – Pre‐eclampsia as defined by trial authors
    – Placental abruption
    – Overweight (BMI >25 and <30)
    – Obesity (BMI >30)
  - Biochemical status:
    – Anaemia (Haemoglobin of <109 g/L)
    – Iron deficiency anaemia
- Foetal outcomes
  - Morbidity
    – Congenital anomalies
- Newborn outcomes
  - Morbidity
    – Low birthweight (<2,500 g)
    – Preterm birth (<37 weeks gestation)
    – Small‐for‐gestational age (World Health Organisation)
    – Macrosomia (birthweight >4,000 g)
  - Anthropometry
    – Birthweight (g)
    – Birth length (cm)
    – Head circumference (cm)
    – Child outcomes
  - Morbidity
    – Stunting (<−2 *Z* score for height for age)
    – Wasting (<−2 *Z* score for weight for height)
    – Underweight (<−2 *Z* score for weight for age)
  - Development outcomes (different scales for psychomotor development, cognitive development, attention, memory, language)
  - Respiratory disease
  - Allergic disease
  - Anaemia:
    – Haemoglobin concentration
    – Iron deficiency anaemia.

Studies were excluded if they have not reported the outcomes mentioned above.

#### 3.1.5 Duration of follow‐up

We will include all participants in eligible studies that had outcomes of interest measured. There will be no restrictions based on duration of exposure, duration of follow‐up, or timing of outcome measurement. If outcomes are reported at multiple time point of follow, we will include outcome based on definitions of outcomes (e.g., neonates 90–28 days vs. infants 0–12 months, etc.). If the time of follow up is not clearly given, we will contact the authors for the same. For childhood and adulthood outcomes, we will include the outcome at the longest follow up.

#### 3.1.6 Types of settings

We will include studies from LMICs. These countries are defined as those with a gross national income (GNI) per capita of USD 1,005 or less in 2016 and lower middle‐income economies are those with a GNI per capita between USD 1,006 and 3,955 in 2016 (World Bank 2017).

### 3.2 Search strategy

We will not impose any restrictions, for example, language, date publication status, on the literature searches described below. We will also search for any relevant retraction statements and errata for information.

#### 3.2.1 Electronic searches

The search will be performed in the following electronic databases: Cochrane Controlled Trials Register (CENTRAL), MEDLINE, EMBASE, CINAHL, PsycINFO, ERIC, Sociofiles, HMIS (Health Management Information Consortium), CAB Global Health (https://www.cabi.org/publishing‐products/online‐information‐resources/global‐health/), the WHO nutrition databases (http://www.who.int/nutrition/databases/en/), Popline (https://www.popline.org), Epistemonikos (https://www.epistemonikos.org/en/), Social Science Citation Index, Dissertation Abstracts International, and WHO Global Health Index which covers the WHO Regional journals from Latin America (LILACS), Africa (AFRO), and so forth. We will also search the web sites of selected development agencies or research firms (e.g., JOLIS, IDEAS, IFPRI, NBER, USAID, World Bank and Eldis.org). The trials registry Clinicaltrials.gov and WHO’s ICRTP will be searched for ongoing trials. Appendix A

#### 3.2.2 Searching other resources

We will make every effort to contact relevant organisations and experts in the field to identify unpublished or ongoing studies. We will also search for citations at Google Scholar and Web of Sciences. References of included articles, relevant reviews, and annotated bibliographies will be scanned for eligible studies. We will also search for *grey literature on*:

- Nutrition International (NI)
- Global Alliance for Improved Nutrition (GAIN)
- World Food Programme (WFP)
- UNICEF
- Emergency Nutrition Network (ENN)
- International Food Policy and Research Institute (IFPRI)
- WHOLIS (WHO library database)
- WHO Reproductive Health Library.

We will also search the reference section of the previously published included studies and systematic reviews. We will also run citation studies of included studies in Google Scholar and Web of Science and/or Scopus. We will also contact other organisations and individual authors who are known experts in the field of maternal nutrition.

### 3.3 Description of methods used in primary research

For this review, the primary research designs of interest were experimental and quasi‐experimental study designs as well as non‐randomised studies with a control group, including CBA. We will also accept ITS studies with at least three time points before and three time points after the intervention. The studies will include pregnant women.

### 3.4 Criteria for determination of independent findings

Before initiating the synthesis (detailed below), we will ensure that all articles reporting on the same study were appropriately linked. To ensure independence and appropriate combination of outcome constructs, syntheses will be conducted according to the type of interventions specified above. If multi‐arm studies are included, intervention groups will be combined or separated into different forest plots, and we will ensure that there was no double counting of participants. If an outcome is reported in several different metrics, we will perform unit conversions in order to pool the data. We will anticipate differences in the types of literature and therefore will ensure that any analysis took possible sources of dependency into account by grouping papers into studies and ensuring that no double counting of evidence took place when synthesising across studies.

### 3.5 Details of study coding categories

Two review authors will extract data independently and a third review author will check for reliability and resolved any conflict. We will extract the primary data for the study characteristics including details of the populations, setting, socio‐demographic characteristics, interventions, comparators, outcomes and study design in duplicate. We will check primary study data for accuracy. Disagreements will be resolved by discussion or consultation with a third reviewer. The following information was extracted for each included study:

- Background: time period when study took place, type of publication (e.g., full‐text journal article, abstract, conference paper and thesis), study country or countries, funding source(s), and conflicts of interest
- Population and setting: population age and setting
- Methods: Study design, description of study arms, unit of allocation, sample or cluster size per study arm (for individually or cluster randomised trials respectively), start and end date, follow‐up
- Participants: total number randomised/allocated, socio‐demographic data
- Intervention group details: number randomised/allocated to group, description of intervention, duration and follow‐up, timing, delivery of intervention, providers and their training. We described all the study intervention arms in the tables of included studies, however, we only reported the intervention arms that met the review inclusion criteria
- Comparison group details: number randomised to group, description of comparison, duration and follow‐up, timing, providers and their training
- Outcomes: measurement tool, validation of the tool, total number in intervention and comparison groups, change indicated at each time point
- Other information.

### 3.6 Statistical procedures and conventions

#### 3.6.1 Assessment of risk of bias in included studies

Two review authors will independently assess the risk of bias for each included study. We will resolve any disagreements by discussion or by involving a third review author.

For RCTs, including cluster RCTs, we will use the Cochrane Collaboration Risk of Bias tool (Higgins et al., 2011). We will assess the risk of bias according to the following domains. We will justify the categorical risk of bias/study quality judgments (e.g., high, low and unclear) with information directly from the study.

- Random sequence generation
- Allocation concealment
- Blinding of participants and personnel
- Blinding of outcome assessment for each outcome
- Incomplete outcome data
- Selective outcome reporting
- Other bias such as the validity of outcome measure and baseline comparability.

For non‐RCTs CAB, and ITS, we will use EPOC methods (Cochrane Effective Practice & Organisation of Care EPOC, 2017) to assess the risk of bias according to the following domains. We justified the categorical risk of bias/study quality judgments (e.g., high, low and unclear) with information directly from the study.

- Random sequence generation
- Allocation concealment
- Baseline outcome measurements
- Baseline characteristics Incomplete outcome
- Knowledge of the allocated interventions adequately prevented during the study
- Protection against contamination
- Selective outcome reporting
- Other risks of bias.

#### 3.6.2 Measures of treatment effect

We will uploaded the outcome data for each study into the data tables in RevMan to calculate the treatment effects (Cochrane Collaboration, 2014). We will use the risk ratio for dichotomous outcomes. We will use the mean difference (MD) for continuous outcomes reported on the same scale, and the standardised MD (SMD) for continuous outcomes reporting the same outcome but measured on different scales. We will express the uncertainty with 95% confidence intervals (CIs) for all effect estimates. When means and standard deviations (SDs) are not reported, we will use other available data (for example, confidence intervals, *t* values, *p* values) and appropriate methods described in the Cochrane Handbook for Systematic Reviews of Interventions (Higgins et al., 2011) to calculate the means and SDs. Where other available data are not sufficient to calculate SDs, we will contact the study authors. When we are unable to enter the results in either way, we will describe them in the table or enter the data into the “Additional tables” section. We will also consider the possibility and implications of skewed data when analysing continuous outcomes as they can mislead results due to small sample size. We will analyse outcomes from studies with multiple groups in an appropriate way to avoid double counting of participants by adding them to different sub‐groups within same plot. In such a scenario, we will not report the overall pooled estimate and only report sub‐group pooled estimate.

#### 3.6.3 Unit of analysis issues

We will have a number of different outcomes and outcome subcategories. Conceptually, these subcategories cannot be combined (e.g., within the cognitive development, language cannot be combined with intelligence). Therefore, a meta‐analysis will be conducted separately for each outcome. Furthermore, for each outcome, we will separately meta‐analysed different study designs (ITS, RCT, and CBA). We will report all the effect sizes for each outcome and not prioritised any from others.

Where trials have used clustered randomisations, we will anticipate that study investigators would have presented their results after appropriately controlling for clustering effects (e.g., variance inflated standard errors, hierarchical linear models). If it is unclear whether a cluster‐RCT has appropriately accounted for clustering, the study investigators will be contacted for further information. Where appropriate controls for clustering are not used, we will request an estimate of the intra‐class correlation coefficient (ICC). Following this, effect sizes and standard errors are meta‐analysed in RevMan using the generic inverse method (Higgins et al., 2011). They will be combined with estimates from individual‐level trials.

#### 3.6.4 Dealing with missing data

We will contact trial authors to verify key study characteristics and obtain missing numerical outcome data where possible (e.g., when we identify a study as an abstract only). If we did not find a full report even after we contact the study authors, we will list such an abstract as a “study awaiting classification”. If numerical outcome data are missing, such as SDs or correlation coefficients, and we could not obtain these from the study authors, we will calculate them from other available statistics, such as *p* values, according to the methods described in the Cochrane Handbook for Systematic Reviews of Interventions (Higgins et al., 2011).

#### 3.6.5 Assessment of heterogeneity

Statistical heterogeneity will be assessed using *τ* ^2^, *I* ^2^, and significance of the *χ* ^2^ test; we will also assess heterogeneity visually using forest plots. On the basis of the prior theory and clinical knowledge, we expect clinical and methodological heterogeneity in effect sizes in this literature. Therefore, we will attempt to explain any observed statistical heterogeneity using subgroup analysis.

#### 3.6.6 Assessment of reporting biases

If sufficient studies are found, funnel plots will be drawn to investigate any relationship between effect size and study precision. Ten studies are usually considered sufficient to draw a funnel plot. As a direct test for publication bias, we will compare the results extracted from published journal reports with results obtained from other sources (including correspondence). Whilst funnel plot asymmetry may indicate publication bias, this will not inevitably the case, and possible explanations for any asymmetry found will be considered and discussed in the text of the review.

We will prepare a matrix of all studies for each intervention which outlined all the differences in the studies in the intervention, duration, timing, and so forth and examine how to pool them together. Our meta‐analyses were random effects meta‐analyses, given the diverse contexts, participants, interventions, and so forth.

For each comparison, we will descriptively summarise the findings from the contextual factors such as setting, timings of intervention, duration of intervention, people delivering interventions, and so forth to assess their impact on the implementation and effectiveness of each intervention.

#### 3.6.7 “Summary of findings” tables

We will construct “Summary of findings” tables for all of the primary outcomes using the Grading of Recommendations Assessment, Development and Evaluation (GRADE) criteria (Guyatt et al., 2011). These cover consideration of within‐study risk of bias (methodological quality), directness of evidence, heterogeneity, precision of effect estimates and risk of publication bias. We will rate the certainty of evidence for each key outcome as “high”, “moderate”, “low”, or “very low”. The GRADE evidence is described in Table 1 **.** Nonrandomized studies will initially be rated as “low” quality. If there are no serious methodological flaws, we will upgrade the evidence for studies with a large magnitude of effect; presence of a dose response relationships; and effect of plausible residual confounding.

**Table 1:** Quality of evidence, as determined by GRADE criteria

Quality	Table text
Very low	Any estimate of effect is uncertain.
Low	Further research is very likely to have important impact on our confidence in the estimate of effect and is likely to change the estimate.
Moderate	Further research is likely to have an important impact on our confidence in the estimate of effect and ay change the estimate.
High	Further research is very unlikely to change our confidence in the estimate of effect.

We will GRADE and prepare the summary of findings tables on the following primary outcomes:

- Stillbirth defined as baby born with no signs of life at or after 28 weeks' gestation
- Neonatal mortality (death <28 days)
- Infant mortality (deaths between 0 and 12 months)
- Under‐five mortality (deaths between 0 and 59 months)
- Low birthweight (<2,500 g)
- Preterm birth (<37 weeks gestation)
- Mean maternal BMI.

#### 3.6.8 Subgroup analysis and investigation of heterogeneity

We will conduct the following subgroup analyses on primary outcomes when there are a sufficient number of studies in each sub‐group. The following sub‐groups would help in differentiating the impact of nutritional interventions for women based on their nutritional status, geographical location and settings and duration of supplementation. This would aid in implementing interventions for specific population.

1. Nutritional status: Undernourished (BMI <18.5) versus well nourished (BMI >18.5) pregnant women defined based on BMI
2. Region: Africa versus South Asia versus South America and Carribean
3. Duration of supplementation: Whole pregnancy versus second trimester versus third trimester
4. Nutritional status: Normal weight versus overweight versus obese (for interventions on maternal obesity)
5. Location: Rural versus Urban versus mixed.

The subgroup analyses will be conducted using Review Manager 5.3 with a test for interaction. We will use *χ* ^2^ statistical tests to assess subgroup differences. *p *< 0.1 will be considered significant for heterogeneity. We will then assess the potential reason of heterogeneity and see if the effect of intervention might be different in certain populations.

#### 3.6.9 Sensitivity analysis

If numbers permit, sensitivity analyses will be performed on the primary outcomes to consider the impact of the following.

- Allocation concealment (adequate versus inadequate and/or unclear).
- Attrition (<10% vs. ≥10%).
- Imputed inter correlation coefficients that have been derived in different ways.

### 3.7 Treatment of qualitative research

We do not plan to include qualitative research.

## ROLES AND RESPONSIBILITIES

● Content: Zohra Lassi, Rehana Salam, Aamer Imdad, Zulfiqar Bhutta.
● Systematic review methods: Aamer Imdad, Zohra Lassi, Rehana Salam.
● Statistical analysis: Zohra Lassi, Rehana Salam, Aamer Imdad.
● Information retrieval: Deepika Ranjit, Gamael Saint Saint Surin, Zohra Lassi.

## SOURCES OF SUPPORT

Funding for this review came from a grant from the Bill & Melinda Gates Foundation to the Centre for Global Child Health at The Hospital for Sick Children (Grant No. OPP1137750).

## DECLARATIONS OF INTEREST

None to declare.

## PRELIMINARY TIMEFRAME

Approximate date for submission of the systematic review. July 2019.

Zohra S. Lassi will be responsible for updating this review and the review will be update every 2 years after publication date.

## APPENDIX A

### Pubmed Search Strategy

#### Population Strategy: Pregnant Women

“Pregnancy”[Mesh] OR “Gravidity”[Mesh] OR “Pregnant Women”[Mesh] OR “Prenatal Care”[Mesh] OR “Perinatal Care”[Mesh] OR “Prenatal Diagnosis”[Mesh] OR “Obstetrics”[Mesh] OR pregnan*[tiab] OR gravid*[tiab] OR gestat*[tiab] OR (pregnant women[tiab] OR pregnant woman[tiab] OR Periconception[tiab]) OR childbear*[tiab] OR maternity[tiab] OR obstetric*[tiab] OR (prepartum[tiab] OR pre partum[tiab]) OR (antepartum[tiab] OR ante partum[tiab]) OR (prenat*[tiab] OR pre nat*[tiab]) OR (antenat*[tiab] OR ante nat*[tiab]) OR (perinat*[tiab] OR peri nat*[tiab])

#### Dietary Diversity Strategies

(“Diet/methods”[Mesh] OR “Diet/trends”[Mesh] OR “Diet/standards”[Mesh]) OR (“Dietary diversity” OR “diet diversity” OR “Diet quality” OR “Dietary Quality” OR “Dietary Quality Index” OR “Dietary Variety” OR “Diet variety” OR “Dietary Diversity Score” OR “Food Variety Score” OR “Nutritional Diversity” OR “Nutrient diversity” OR “Nutritional Functional Diversity” OR “Nutritional adequacy” OR “Nutrient adequacy” OR “Nutrition adequacy”) OR (Dietary Pattern* OR Diet Pattern* OR Nutritional pattern* OR Nutrition pattern*) **19478**

#### Bep Supplementation

(“Energy Intake”[Mesh] OR “Dietary Proteins”[Mesh] OR protein[tiab] OR energy[tiab] AND supplement*) **53670**

#### Food Distribution Programmes

“Food assistance”[Mesh] OR “Food assistance” OR “Food distribution” OR "Food aid” OR “Nutrition assistance” OR food programme* (You might get more relevant studies if you can add more to this for a more nuanced approach, so if you were using Ovid Medline it might be: ((food* or diet* or micronutrient*) adj2 (programme*)). **2180**

#### Maternal Obesity Interventions

(“Obesity/prevention and control”[Mesh] OR “Weight Gain”[Mesh]) AND “Humans”[Mesh])) **30781**.
